# Adverse childhood experiences and their impact on primary headache patients: a cross-sectional study

**DOI:** 10.3389/fpsyt.2025.1676559

**Published:** 2025-10-23

**Authors:** Alexander Pabón Moreno, Valentina Gonzalez Galindo, Alexandra Hurtado-Ortiz, Maricel Licht-Ardila, Edgar Fabian Manrique-Hernández, Alejandra Mendoza-Monsalve, Angelica Tatiana Pérez-Cárdenas, Ximena Jaely Forero, Andreina Judith Portilla, Federico Silva Sieger, Elkin Llanez Anaya

**Affiliations:** ^1^ Neurological Institute, Hospital Internacional de Colombia – Fundación Cardiovascular de Colombia, Piedecuesta, Colombia; ^2^ Postgraduate Department in Infectious Disease, Universidad de Santander, Bucaramanga, Colombia; ^3^ Neurovascular Sciences Group, Hospital Internacional de Colombia – Fundación Cardiovascular de Colombia, Piedecuesta, Colombia; ^4^ Universidad de Santander, Faculty of Medical Sciences and Health, School of Medicine, Neuroscience Group, Bucaramanga, Colombia; ^5^ Department of Epidemiology, Hospital Internacional de Colombia – Fundación Cardiovascular de Colombia, Piedecuesta, Colombia

**Keywords:** adverse childhood experiences, headache disorders, neurology, childhood trauma, risk factors

## Abstract

**Introduction:**

Adverse Childhood Experiences (ACEs), including abuse and household dysfunction, can have lasting effects on development and health, increasing risks for chronic diseases and mental health issues.

**Objective:**

to estimate the prevalence of ACEs in this population and to determine the factors associated with these experiences.

**Methods:**

A cross-sectional study was conducted at a Colombian high-complexity institution, including adults with primary headaches according to ICHD-3 criteria. Statistical analysis involved bivariate comparisons and multivariate logistic regression, with goodness-of-fit assessed using the Hosmer and Lemeshow test. All analyses were performed using Stata 16.

**Results:**

138 patients with primary headaches were included, 77.54% reported experiencing some form of ACEs, with 34.06% having scores of 4 or higher. Physical abuse was the most common ACE (9.13%). Women had a higher probability of reporting ACEs (OR: 8.613, 95% CI: 1.006-73.776, p = 0.049). Those with severe disability (MIDAS score) were less likely to report severe ACEs (OR: 0.293, 95% CI: 0.096-0.899, p = 0.032).

**Conclusion:**

This study demonstrates a strong relationship between adverse childhood experiences and primary headaches, highlighting the need to incorporate childhood trauma assessment into neurological practice.

## Introduction

Adverse Childhood Experiences (ACEs) are a set of interrelated negative events where the lack of individual, family, and/or environmental resources to adequately address them can transform these events into lasting traumatic experiences, directly affecting children and adolescents before the age of 18 (1). Exposure to various forms and repeated instances of abuse is linked to higher risks of severe mistreatment and psychological effects (2). These include a wide range of early life traumatic events, such as emotional, sexual, and physical abuse, as well as various forms of household dysfunction. In general terms, adverse experiences are more common in children under the age of six than in older children (3). Studies have revealed that nearly all children between the ages of 18 and 71 months (98.1%) have experienced at least one adverse event, and 50.5% have encountered four or more (4). Which can significantly burden individuals negatively, affecting their quality of life, leading to risky behaviors, and contributing to the development of chronic non-communicable diseases in adulthood (5).

**Figure 1:**
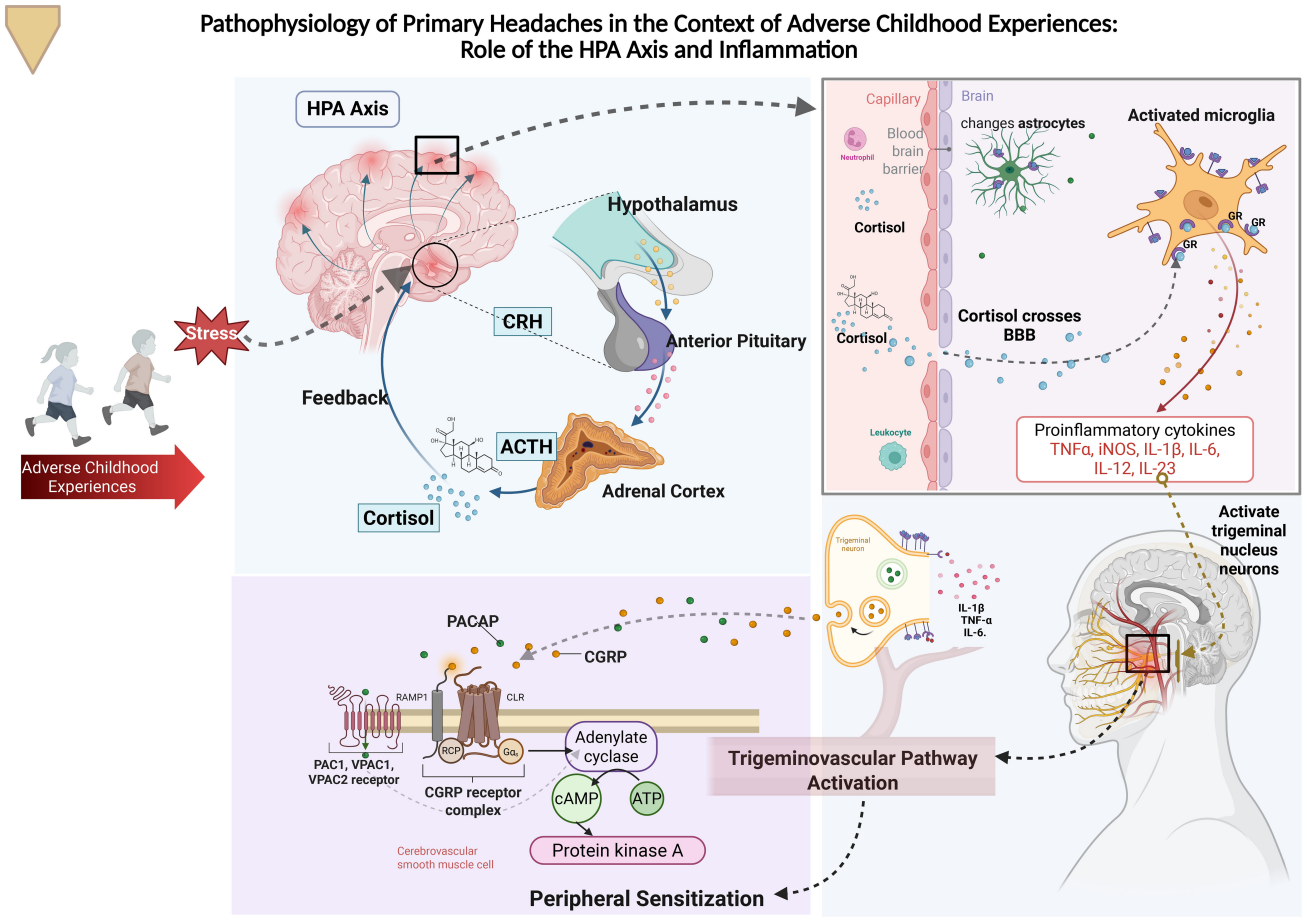
Adverse childhood experiences (ACEs) chronically activate the HPA axis, elevating cortisol, adrenaline, cytokines, and CGRP, which affect neuronal development and emotional regulation. This dysfunction can predispose individuals to the development of primary headaches, such as migraine, tension-type headache, and trigemino-autonomic headaches, by sensitizing the trigeminovascular system. Additionally, chronic inflammation and altered stress response increase the risk of physical and emotional illnesses, as well as premature death. HPA axis, Hypothalamic-Pituitary-Adrenal axis; CRH, Corticotropin-Releasing Hormone; ACTH, Adrenocorticotropic Hormone; IL-1β, Interleukin-1 beta; TNF-α, Tumor Necrosis Factor alpha; IL-6, Interleukin-6; PACAP, Pituitary Adenylate Cyclase-Activating Polypeptide; CGRP, Calcitonin Gene-Related Peptide; PAC1, PACAP Receptor 1; VPAC1, Vasoactive Intestinal Peptide/PACAP Receptor 1; VPAC2, Vasoactive Intestinal Peptide/PACAP Receptor 2; RCP, Receptor Component Protein; Gαs, Stimulatory G Protein alpha subunit; cAMP, Cyclic Adenosine Monophosphate; ATP, Adenosine Triphosphate.

The pathophysiology of ACEs is related to the impact of toxic stress on the hypothalamic-pituitary-adrenal (HPA) axis, which develops during childhood and regulates the production of stress hormones. Toxic stress continuously stimulates the HPA axis, leading to a sustained increase in cortisol, adrenaline, and cytokines, generating a chronic state of stress and epigenetic changes that alter the regulation of the gene responsible for cortisol production, raising its levels even in non-stressful situations. This also activates microglia, which intensifies neural pruning during development, affecting neuronal connections crucial for emotional regulation and executive function (6). As a result, there is suboptimal development of physical, social, emotional, and cognitive skills, as well as an increased risk of physical and emotional diseases and premature death due to systemic inflammation caused by toxic stress (7) (**Figure 1**).

ACEs are associated with early mortality and morbidity (8). Among the risks associated with ACEs, a significant increase in various physical and mental health problems has been observed. In the physical domain, there is a fourfold increase in the risk of developing diabetes, a twofold increase in obesity, a twofold increase in acute myocardial infarction, and a twofold increase in cancer (9). Regarding mental health, the association between early life stress, the functioning of the HPA axis, and psychiatric conditions is nuanced (10). Studies have shown there is an increased risk of suicide attempt (12 times), depression (5 times), and addictions (alcohol, smoking, substance abuse). Additionally, ACEs are associated with poorer academic and work performance, which negatively impacts individuals’ quality of life and increases healthcare costs (9, 11).

Given that headaches represent one of the most frequent complaints in primary care, with studies showing that 52% of the global population has experienced a headache disorder in a year; 4.6% suffer from headaches for 15 or more days per month, 14% report migraines, and 26% report tension headaches (12), it is crucial to analyze the relationship between adverse childhood experiences and their effects on headaches (13). This chronic condition affects individuals regardless of demographic or economic factors, presenting as a debilitating symptom that deteriorates quality of life and leads to significant economic costs (14). The frequent occurrence of headaches and the fear of new episodes negatively impact family life, social relationships, and work (15).

Multiple studies have shown a connection between adverse childhood experiences and the development of chronic migraines in adulthood. A study published in Headache: The Journal of Head and Face Pain found that people with chronic migraines reported a higher incidence of emotional, physical, and sexual abuse compared to those without migraines. The study suggests that childhood trauma may predispose certain individuals to chronic migraines due to long-lasting neurobiological and psychological changes that affect pain perception and the response to stress (16). Therefore, further research is being pursued on the link between adverse childhood experiences and primary headaches. In this context, an analysis of ACEs will be conducted among patients with primary headaches treated at the International Hospital of Colombia. The study aims to estimate the prevalence of ACEs in this population and identify the factors associated with these experiences.

## Methods

A cross-sectional analytical study was conducted on patients from the Neurology Service, associated with the Primary Headache Integrated Practice Unit at a high-complexity institution in Colombia, during the year 2023 (January-December). The inclusion criteria were age 18 years or older with a diagnosis of primary headache, according to the diagnostic criteria of The International Classification of Headache Disorders (ICHD-3), updated until 2019 (17), and those who completed the informed consent form agreeing to participate in the study. Exclusion criteria included individuals with systemic inflammatory disorders, including rheumatic and autoimmune inflammatory disorders, active malignancy, ongoing chronic infection, pregnancy or lactation at the time of the study, previous neurological conditions such as epilepsy, neuromuscular and metabolic diseases, brain tumors, and diagnosis of terminal illnesses.

Demographic variables included age, sex, marital status, education level, and socioeconomic status. Additionally, the classification of primary headache, the presence of non-communicable chronic diseases, exacerbating factors, pain last, headache frequency, age of onset of symptoms and scores on various scales and questionnaires were recorded, such as the Beck Depression Inventory-II (BDI-II Scale) for measuring depression severity (18), the Adverse Childhood Experiences Questionnaire (19), the Headache Impact Test (HIT-6 Scale) for measuring the impact of headaches on functionality (20, 21), the Visual Analog Scale for Pain (VASP) (21), the Pittsburgh Sleep Quality Index (PSQI) (22), and the Migraine Disability Assessment scales (MIDAS) for measuring the degree of disability secondary to migraine (23).

Information was collected using REDCap (Research Electronic Data Capture) software, ensuring data anonymity and privacy. The diagnostic interview was conducted by a multidisciplinary team consisting of a neurologist specialized in headache disorders, a psychiatrist (MDR), a family medicine resident, and nursing staff, all trained in the application of standardized instruments for headache and psychiatric evaluation. Patients were consecutively recruited during routine consultations at the Neurology Service and the Primary Headache Integrated Practice Unit at the International Hospital of Colombia. Recruitment was based on patient attendance during the study period, without randomization. The questionnaire used to assess ACEs included 10 categories of adversities grouped into three main areas: abuse (physical, emotional, and sexual), neglect (physical and emotional), and family dysfunction (mental health problems, substance abuse, incarceration of a family member, domestic violence, and parental separation or divorce) (19).

### Statistical analysis

Qualitative variables were expressed as proportions and percentages, while quantitative variables were presented as medians and interquartile ranges because of its non-normal distribution. Also, a bivariate statistical analysis was conducted between using the Chi-square test or Fisher’s exact test, and quantitative and qualitative variables were assessed with the Kruskal-Wallis test. The outcome variable was categorized into non-adverse experiences, mild adverse experiences (score 1-3), and complex trauma (score >=4). A multivariate analysis was performed using logistic regression, with the presence or absence of complex trauma as the outcome, to evaluate the related variables in this population. The goodness-of-fit was then assessed using the Hosmer and Lemeshow test. All analyses were conducted using Stata software, version 16.

### Ethical statement

The study was conducted in accordance with national and international ethical standards, including the principles outlined in the Declaration of Helsinki. Ethical approval was obtained from the Ethics Committee of the Fundación Cardiovascular de Colombia (CEI-2024-07461). All data collected were handled in strict compliance with personal data protection laws.

## Results

A total of 138 patients with primary headache were evaluated, of which 77.54% presented some form of ACE, and 34.06% scored 4 or higher. In the analyzed population, 89.86% were women, with statistically significant differences observed across groups. The median age ranged between 40 and 44 years, with the highest median age observed in the group without ACE (median: 44 years, IQR: 33-57). In all three groups, over 80% of the subjects were from urban areas, and the socioeconomic status was similarly distributed, with strata 2 and 6 predominating. Marital status was evenly distributed, with no statistically significant differences. Regarding medical history, hypertension was more prevalent in the group with ACE 1-3 (18.33%), although no statistically significant differences were found between the groups, and the prevalence of diabetes and depression was below 3% (**Table 1**).

**Table 1:** Sociodemographic and medical history characteristics of patients with primary headache according to childhood adverse experiences questionnaire result.

Variable	None EAI n=31 (22.46%)	EAI 1–3 n=60 (43.48%)	EAI >=4 n=47 (34.06%)	P-value
Socio-demographic				
Age (years) *	44 (33–57)	42 (34.5-51)	40 (32 – 49)	0.493
Sex				0.030
Male	8 (25.81)	5 (8.33)	1 (2.13)	
Female	23 (74.19)	55 (91.67)	46 (97.87)	
Area of origin				0.927
Rural	6 (19.35)	10 (16.67)	9 (19.15)	
Urban	25 (80.65)	50 (83.33)	38 (80.85)	
Educational level				0.599
None	9 (29.03)	14 (23.33)	9 (19.15)	
Some educational level	22 (70.97)	46 (76.67)	38 (80.85)	
Socioeconomic stratum				0.382
1	1 (3.22)	4 (6.67)	6 (12.77)	
2	12 (38.70)	29 (48.33)	17(36.17)	
3	4 (12.90)	12 (20.00)	9 (19.15)	
4	3 (9.67)	2 (3.33)	1 (2.13)	
6	11 (35.48)	13 (27.67)	14 (29.79)	
Marital status				0.596
Without permanent partner	15 (48.39)	30 (50.00)	19 (40.43)	
With permanent partner	16 (51.61)	30 (50.00)	28 (59.57)	
Medical history				
Hypertension				0.267
Yes	3 (9.86)	11 (18.33)	4 (8.51)	
No	28 (90.32)	49 (81.67)	43 (91.49)	
Diabetes				0.462
Yes	0 (0.00)	3 (5.00)	2 (4.26)	
No	31 (100)	57 (95.00)	45 (95.74)	
Depression				0.731
Yes	0 (0.00)	1 (1.67)	1 (2.13)	
No	31 (100.00)	59 (98.33)	46 (97.87)	

*Median-Interquartile range (IQR).

In the analysis of clinical characteristics of patients with primary headaches in relation to ACE, several notable trends emerged. The age of symptom onset revealed that patients with 1 to 3 ACE events reported a higher prevalence of headaches in early childhood and adolescence compared to those with no ACE or 4 or more events. Headache frequency was predominantly chronic across all groups, with no significant differences in episodic frequency between them. Patients with a greater number of ACEs tended to experience longer-lasting headaches, often exceeding 4 hours in duration. Regarding triggering factors, stress was the most frequently reported, followed by sleep patterns. Body mass index (BMI) and the VASP did not show significant differences among the groups. The most common headache types were chronic and episodic migraines, with no substantial variation in their distribution across the groups. Patients with more severe ACEs exhibited greater disability according to the MIDAS scale and poorer sleep quality as measured by the PSQI. Scores on the BDI-II indicated a higher prevalence of depression in the group with more severe ACEs, and the IDARE scale revealed higher levels of trait anxiety in these patients (**Table 2**).

**Table 2:** Clinical characteristics of patients with primary headache according to childhood adverse experiences questionnaire result.

Variable	None EAI n=31 (22.46%)	EAI 1–3 n=60 (43.48%)	EAI >=4 n=47 (34.06%)	P-value
Age of onset of symptoms (years)				0.131
Early Childhood (0-5)	1 (3.23)	1 (1.67)	0 (0,00)	
Childhood (6-11)	2 (6.45)	16 (26.67)	9 (19.15)	
Adolescence (12-18)	6 (19.35)	13 (21.67)	9 (19.15)	
Youth (19-26)	2 (6.45)	8 (13.33)	9 (19.15)	
Adulthood (27-59)	17 (54.84)	22 (36.67)	17 (36.17)	
Older Adult (>60)	3 (9.68)	0 (0.0)	3 (6.38)	
Headache frequency (attacks/month)				0.901
Low-frequency Episodic (< 8)	7 (22.58)	12 (20)	8 (17.02)	
High-frequency Episodic (8- 15)	9 (29.03)	22 (36.67)	15 (31.91)	
Chronic (> 15)	15 (48.39)	26 (43.33)	24 (51.06)	
Pain last				0.148
1 second to 10 minutes	0 (0.0)	1 (1.69)	0 (0.00)	
10 minutes to 30 minutes	2 (6.67)	0 (0.0)	0 (0.00)	
30 minutes to 4 hours	9 (30)	9 (15.25)	12 (25.53)	
More than 4 hours	10 (33.33)	28 (47.46)	18 (38.30)	
Continuous	9 (30)	21 (35.59)	17 (36.17)	
Triggering or exacerbating factors				0.419
Stress	12 (38.71)	28 (47.46)	24 (51.06)	
Sleep pattern	5 (16.13)	13 (22.03)	11 (23.40)	
Others	9 (29.03)	10 (16.95)	4 (8.51)	
Unspecified	5 (16.13)	8 (13.56)	8(17.02)	
BMI*	26.5 (24.2-28.2)	26.7 (24.7-29)	27.3 (24.3-29.7)	0.721
VASP (0-10)*	8 (6-9)	8 (8-10)	9(7-10)	0.253
Type of primary headache				0.269
Episodic Low-Frequency Migraine with Aura	3 (9.68)	3 (5)	4 (8.51)	
Episodic Low-Frequency Migraine without Aura	3 (9.68)	11 (18.33)	7 (14.89)	
Episodic High-Frequency Migraine with Aura	3 (9.68)	11 (18.33)	5 (10.64)	
Episodic High-Frequency Migraine without Aura	2 (6.45)	12 (20)	8 (17.02)	
Chronic Migraine	12 (38.71)	18 (30)	20 (42.73)	
Episodic Low-Frequency Tension-Type Headache	2 (6.45)	0 (0.00)	0 (0.00)	
Episodic High-Frequency Tension-Type Headache	2 (6.45)	1 (1.67)	2 (4.26)	
Chronic Tension-Type Headache	2 (6.45)	3 (5)	1 (2.3)	
Trigeminal Autonomic Headache	2 (6.45)	1 (1.67)	0 (0.00)	
MIDAS				0.324
Severe Disability	8 (25.81)	15 (25.42)	5 (10.87)	
Mild Disability	2 (6.45)	8 (13.56)	4 (8.70)	
Moderate Disability	3 (9.68)	6 (10.17)	9 (19.57)	
No Disability	18 (58.06)	30 (50.85)	28 (60.87)	
HIT-6				
Some impact	2 (6.45)	4 (6.90)	4(8.70)	0.342
Substantial impact	2 (6.45)	8 (13.79)	2(4.35)	
Severe impact	23 (74.19)	41 (70.69)	39(84.78)	
Little or no impact	4 (12.90)	5 (8.62)	1(2.17)	
BDI-II				
Minimal depression	19 (70.37)	39 (57.69)	17(43.59)	0.403
Mild Depression	3 (11.11)	12 (23.08)	10(25.64)	
Moderate Depression	3 (11.11)	6 (11.54)	9(23.08)	
Severe Depression	2 (7.41)	4 (7.69)	3(7.69)	
BDI-II (continuous)	9 (4-14)	11 (7)(17.5)	16 (11- 25)	0.004
IDARE				0.031
Low	6 (23.08)	4 (7.84)	0(0.00)	
Moderate	11 (42.31)	23 (45.10)	19(50)	
High	9 (34.62)	24 (47.06)	19(50)	
PSQI				0.010
Poor sleep quality	18 (69.23)	34 (65.38)	36(92.31)	
No sleep problems	8 (30.77)	18 (34.62)	3 (7.69)	

VASP, Visual Analog Scale for Pain; MIDAS, Migraine Disability Assessment; HIT-6, Headache Impact Test; BDI-II, Beck Depression Inventory; IDARE, Scale for Trait Anxiety Detection; PSQI, Pittsburgh Sleep Quality Index.

**Figure 2:**
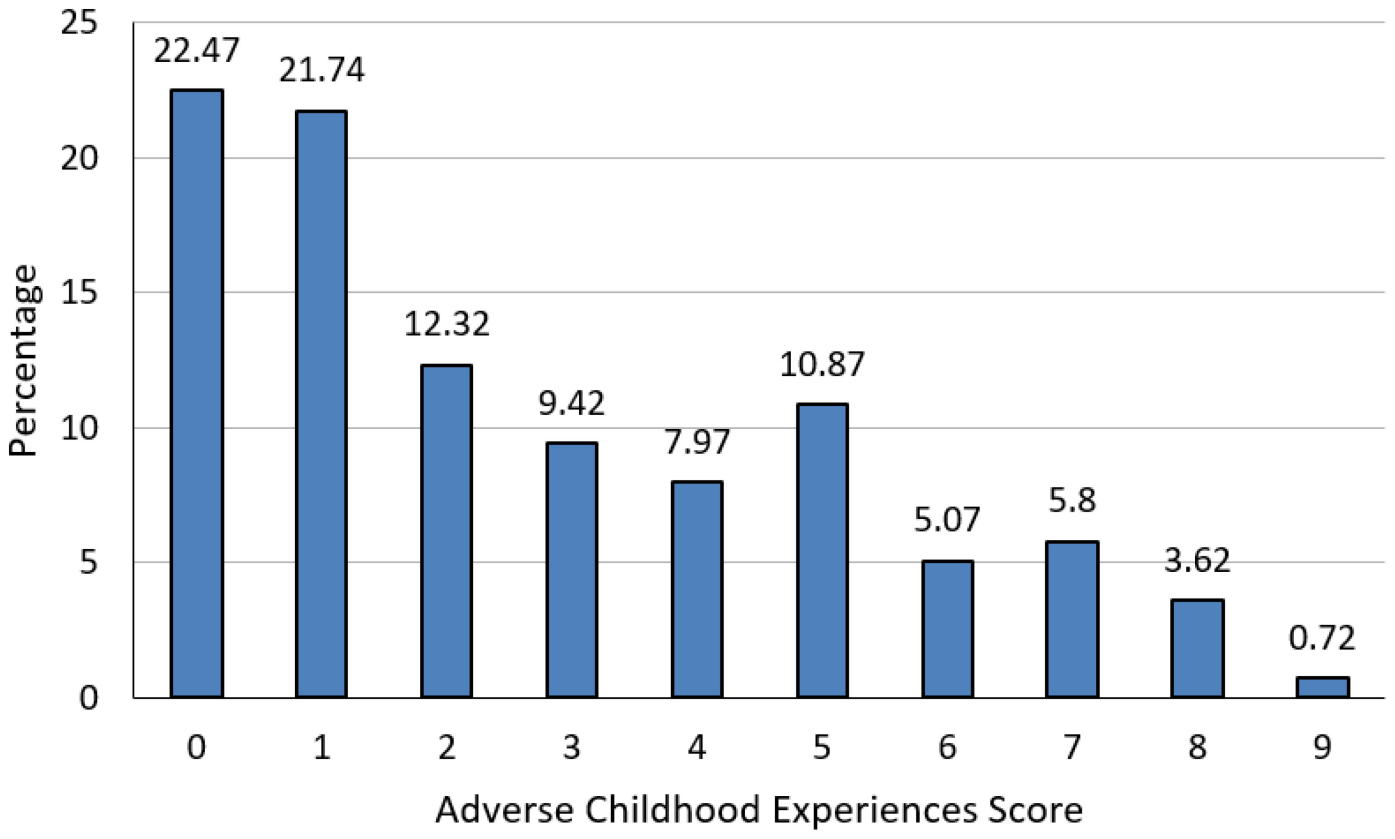
Distribution of the study population according to adverse childhood experiences questionnaire score.

Additionally, it was observed that 22.46% of the population did not report any ACEs. Among those who did, 21.4% reported a single event, while 10.87% experienced five events (**Figure 2**).

**Figure 3:**
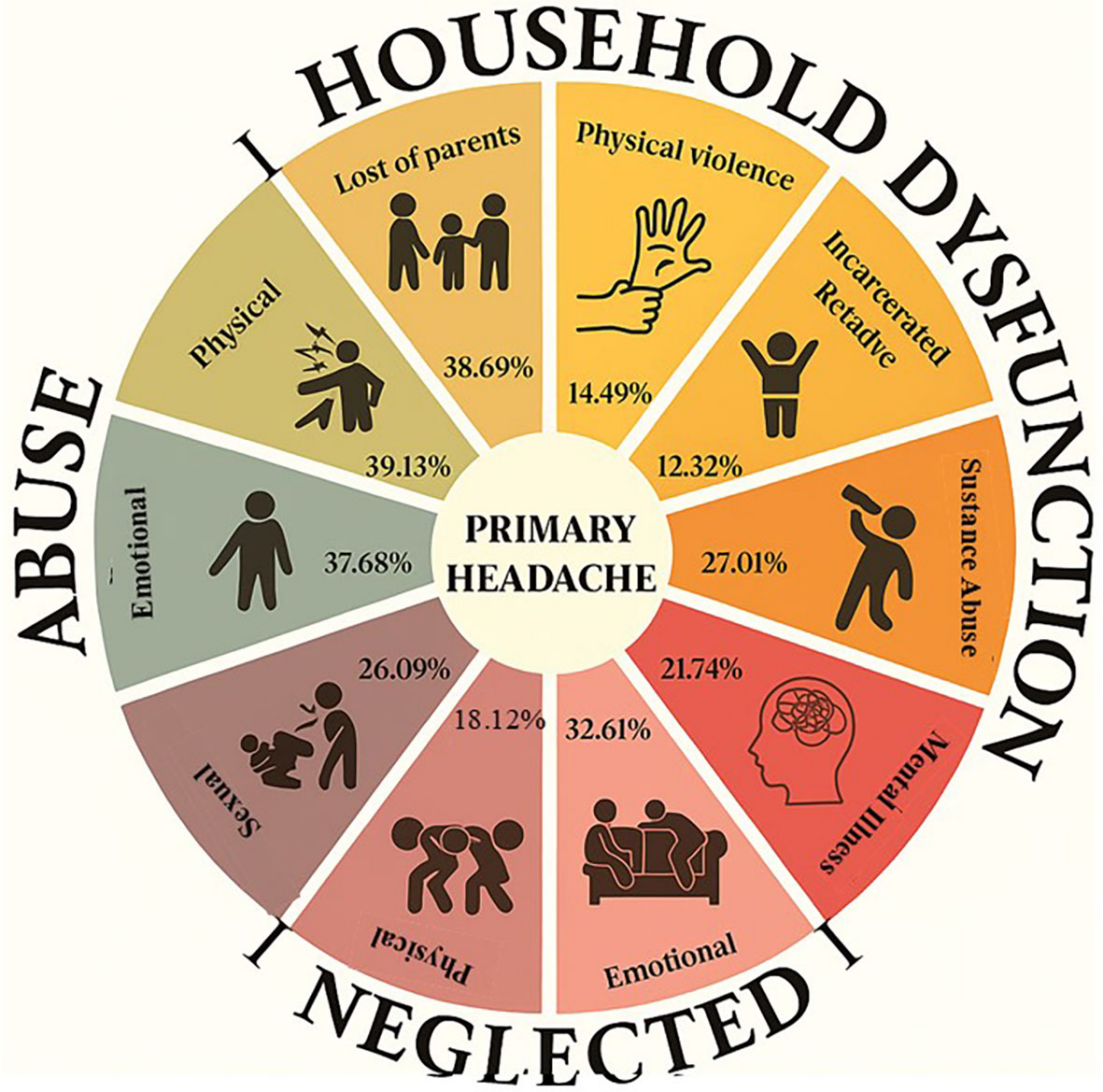
Distribution of reported adverse experiences.

Among the participants, physical abuse was the most prevalent adverse experience, affecting 39.13% of the population. Regarding abandonment, the emotional aspect was the most reported, with 32.61%. Concerning family dysfunction, the loss of parents affected 38.69% of individuals. Other adverse experiences included substance abuse, which impacted 27.01% of the participants. These data are illustrated in **Figure 3**.

In the multivariable analysis, significant variables associated with adverse childhood experiences were identified. Notably, gender was found to be a significant factor (OR: 8.613, 95% CI: 1.006-73.776, p = 0.049), indicating that women are more likely to have experienced these adverse events in childhood compared to men. Additionally, the MIDAS score was also significant (OR: 0.293, 95% CI: 0.096-0.899, p = 0.032), showing that individuals with severe disability have a lower probability of having experienced severe adverse events compared to those with mild disability (see **Table 3**). The model’s goodness of fit, assessed using the Hosmer-Lemeshow test, yielded a p-value of 0.2339, indicating that the model fits the data well (**Table 3**).

**Table 3:** Multivariate analysis of factors associated with adverse childhood experiences.

Variable	OR	SD	p value	95% CI
Sex (Female)	8.613	94.383	0.049	1.006-73.776
Age	0.980	0.0157	0.212	0.950-1.011
MIDAS questionnaire				
Mild Disability	0.824	0.5767	0.782	0.209-3.248
Moderate Disability	1.240	0.6873	0.698	0.418-3.675
Severe Disability	0.293	0.1676	0.032	0.096-0.899
HIT-6				
Moderate	6.100	78.005	0.157	0.498-74.783
Substantial	1.736	23.409	0.683	0.123-24.406
Severe	6.156	67.830	0.099	0.710-53.361

OR, Odds Ratio; MIDAS, Migraine Disability Assessment; HIT-6, Headache Impact Test; SD, Standard Deviation; CI, Confidence Interval.

## Discussion

This study aimed to explore the prevalence and impact of ACEs among patients with primary headaches treated at the Primary Headache Center of Excellence at the International Hospital of Colombia. The findings highlight the significant presence of ACEs in this population, with 77.54% of patients reporting at least one adverse experience and 34.06% reporting four or more adverse events, which can be considered as complex trauma. This high prevalence aligns with existing literature that indicates a strong correlation between childhood trauma and chronic health conditions, including headaches (24).

One of the most notable findings of this study is the gender discrepancy in the prevalence of ACEs, with a higher percentage of women reporting these experiences. Although our sample predominantly consists of women, it was observed that they were more likely than men to report ACEs. This finding aligns with previous research that suggests women are more vulnerable to experiencing ACEs and subsequently developing chronic conditions such as migraines and other primary headache disorders (25). This gender disparity may be attributed to a combination of biological, psychological, and sociocultural factors that not only predispose women to greater exposure to ACEs but may also make them more susceptible to their long-term negative effects. Recent longitudinal studies confirm that women show higher vulnerability to the negative effects of early adverse environments, while also benefiting more from protective factors such as social support and school belonging (26). Moreover, systematic reviews emphasize that gender-specific approaches are necessary, as certain types of ACEs differentially impact men and women (27). These findings support our results, in which female gender emerged as a significant predictor of ACEs (OR: 8.613, p = 0.049).

This study revealed a complex relationship between the severity of ACEs and the clinical characteristics of headaches (24). It is thus how those with severe ACEs (scores ≥ 4) tended to report longer-lasting headaches and a higher prevalence of associated symptoms, such as poor sleep quality and depression (28). However, these patients were less likely to report severe disability according to the MIDAS scale. This finding, which might seem contradictory, suggests that while ACEs exacerbate certain aspects of the headache experience, they may also contribute to greater pain tolerance or different coping mechanisms in some patients (29). It should be noted that headache duration must be interpreted with caution, as it represents a diagnostic criterion that differentiates among various headache types. According to the ICHD-3 classification, migraine attacks typically last between 4 and 72 hours, whereas tension-type headaches may extend from 30 minutes to 7 days (17).

Regarding the association between ACEs and mental health, this study also highlighted the significant impact of ACEs on the mental health of patients with headaches (30). Higher ACE scores were associated with worse outcomes on the Beck Depression Inventory (BDI-II) and the IDARE scale for anxiety. These findings are reinforced by recent longitudinal evidence showing that anxiety and depressive symptoms significantly mediate the relationship between ACEs and the development of persistent or recurrent pain in youth, identifying them as key therapeutic targets. Furthermore, an umbrella review demonstrated that ACEs are associated with a 66% increased risk of anxiety and depression in adulthood. In the specific context of primary headaches, ACEs have been identified as important risk factors for disease burden and chronicity (31).

A study conducted by López et al. in 2021 incorporated mindfulness training and EMDR (Eye Movement Desensitization and Reprocessing) therapy for adolescents with multiple adverse experiences, which significantly reduced PTSD-related symptoms and increased attention/awareness-related outcomes in adolescent girls with multiple ACEs (32), in 2022 they extended these prior findings, revealing significant epigenetic changes, suggesting that such interventions can effectively address the psychological burden in adolescents with a history of multiple adverse experiences (33).

The research conducted at the Primary Headache Center of Excellence has clinical implications and projects future interventions based on the high prevalence of ACEs among patients with primary headaches. This suggests that the evaluation of childhood trauma should be an integral part of the diagnostic and therapeutic process in headache management. Given the significant associations between ACEs, headache severity, and mental health outcomes, clinicians should consider multidisciplinary approaches that include psychological support and interventions aimed at addressing the long-term effects of childhood trauma. Additionally, future research must focus on longitudinal studies to better understand the causal pathways between ACEs and primary headache disorders. Moreover, exploring the effectiveness of trauma-informed care models in improving outcomes for patients with headaches and a history of ACEs could provide valuable insights for clinical practice.

This cross-sectional study has limitations. Most notably, because of its cross-sectional design, no firm causal link can be established between ACEs and primary headaches, and the observed associations should be interpreted as correlations rather than causal effects. Additionally, its setting in a specialized center may introduce selection bias, and because a non-probabilistic convenience sample was used, the study population may not be fully representative of the broader primary headache population in Colombia. The reliance on self-reported data could lead to recall bias. The sample was predominantly female, which could limit the generalizability to male patients; however, this sex distribution is in line with epidemiological data showing that migraine affects women nearly twice as often as men globally (12), and similarly in Latin America, where prevalence estimates in Colombia are roughly 13.8 % in women versus 4.8 % in men (34). Some predictors, such as sex, showed very wide confidence intervals, reflecting limited statistical power and reduced stability of the estimates; therefore, these associations should be interpreted with caution and considered exploratory. To better understand the temporal relationship between ACEs and headaches, future longitudinal studies with more diverse populations are needed.

Interestingly, patients with severe disability according to MIDAS were less likely to report severe ACEs, a finding that seems counterintuitive. Several explanations may account for this result. First, recall or reporting bias cannot be excluded, as patients with greater functional impairment may prioritise reporting their current symptom burden over early-life adversities. Second, resilience and coping mechanisms acquired in response to adversity could mitigate the perceived impact of ACEs on headache-related disability. Third, clinical factors such as chronicity, treatment adherence, or comorbid conditions may exert a stronger influence on disability scores than childhood experiences in this subgroup. This unexpected association should therefore be interpreted cautiously and warrants further investigation in prospective studies.

Nevertheless, this study has several notable strengths. It is one of the few studies to explore the relationship between Adverse Childhood Experiences (ACEs) and headaches in a Latin American population, offering valuable insights into an under-researched region. Moreover, the inclusion of multiple validated tools to assess both ACEs and the clinical aspects of headaches, such as MIDAS, BDI-II, and PSQI, provides a comprehensive and accurate evaluation of these variables. The use of a multivariate approach further strengthens the internal validity by controlling for potential confounding factors.

## Conclusion

This study demonstrates the strong relationship between ACEs and primary headaches, underscoring the need to integrate childhood trauma assessment into neurological practice. Women, who show greater vulnerability to ACEs, require specific interventions that address both the neurological and psychological aspects of these experiences.

## Data Availability

The raw data supporting the conclusions of this article will be made available by the authors, without undue reservation.
